# Low-input breeding potential in stone pine, a multipurpose forest tree with low genome diversity

**DOI:** 10.1093/g3journal/jkaf056

**Published:** 2025-03-12

**Authors:** Sanna Olsson, David Macaya-Sanz, Carlos Guadaño-Peyrot, Sara Pinosio, Francesca Bagnoli, Camilla Avanzi, Giovanni G Vendramin, Neus Aletà, Ricardo Alía, Santiago C González-Martínez, Sven Mutke, Delphine Grivet

**Affiliations:** Institute of Forest Sciences (ICIFOR-INIA), Consejo Superior de Investigaciones Cientificas, Madrid 28040, Spain; Institute of Forest Sciences (ICIFOR-INIA), Consejo Superior de Investigaciones Cientificas, Madrid 28040, Spain; Institute of Forest Sciences (ICIFOR-INIA), Consejo Superior de Investigaciones Cientificas, Madrid 28040, Spain; Higher-Technical School of Agricultural Engineering, University of Valladolid, Palencia 47002, Spain; Institute of Biosciences and Bioresources, National Research Council (CNR), Sesto Fiorentino 50019, Italy; Institute of Biosciences and Bioresources, National Research Council (CNR), Sesto Fiorentino 50019, Italy; Institute of Biosciences and Bioresources, National Research Council (CNR), Sesto Fiorentino 50019, Italy; Institute of Biosciences and Bioresources, National Research Council (CNR), Sesto Fiorentino 50019, Italy; Fruit Growing Program, Institute of Agrifood Research and Technology (IRTA), Torre Marimon, Caldes de Montbui 08140, Spain; Multifunctional Forest Management Program, Forest Science and Technology Centre (CTFC), Solsona 25280, Spain; Institute of Forest Sciences (ICIFOR-INIA), Consejo Superior de Investigaciones Cientificas, Madrid 28040, Spain; UMR BIOGECO, INRAE, University of Bordeaux, Cestas 33610, France; Institute of Forest Sciences (ICIFOR-INIA), Consejo Superior de Investigaciones Cientificas, Madrid 28040, Spain; Institute of Forest Sciences (ICIFOR-INIA), Consejo Superior de Investigaciones Cientificas, Madrid 28040, Spain

**Keywords:** Mediterranean stone pine, pine nuts, clonal identification, genomic prediction, SNP-array

## Abstract

Stone pine (*Pinus pinea* L.) is an emblematic tree species within the Mediterranean basin, with high ecological and economic relevance due to the production of edible nuts. Breeding programmes to improve pine nut production started decades ago in Southern Europe but have been hindered by the near absence of polymorphisms in the species genome and the lack of suitable genomic tools. In this study, we assessed new stone pine's genomic resources and their utilization in breeding and sustainable use, by using a commercial SNP-array (5,671 SNPs). Firstly, we confirmed the accurate clonal identification and identity check of 99 clones from the Spanish breeding programme. Secondly, we successfully estimated genomic relationships in clonal collections, an information needed for low-input breeding and genomic prediction. Thirdly, we applied this information to genomic prediction for the total number of cones unspoiled by pests and their weight measured in 3 Spanish clonal tests. Genomic prediction accuracy depends on the trait under consideration and possibly on the number of genotypes included in the test. Predictive ability (*r*_y_) was significant for the mean cone weight measured in the 3 clonal tests, while solely significant for the number of cones in one clonal test. The combination of a new SNP-array together with the phenotyping of relevant commercial traits into genomic prediction models, proved to be very promising to identify superior clones for cone weight. This approach opens new perspectives for early selection.

## Introduction

Traditionally improving the overall performance of a forest tree population (i.e. maximizing the genetic gain of traits of interest), while maintaining its genetic diversity, relies on recurrent cycles of activities including selection, breeding, and genetic testing (White 2004). These programmes would benefit on the high-throughput genomic and phenotypic characterization of individuals from common gardens and/or natural populations (Neale and Kremer 2011). This combination is appealing to understand the genetic architecture of traits (i.e. the genotype–phenotype relationships), to monitor adaptive gene diversity more precisely, and to develop genomics-based applications, such as marker-assisted selection or genomic selection, which are useful both in evolutionary genetics and breeding (Neale and Kremer 2011; McGaugh *et al*. 2021).

In contrast to traditional breeding, low-input breeding strategies focus on reducing the investment of time and material resources, and are appropriate for species of low importance, both now and in the foreseeable future (Namkoong *et al*. 1980). Various genomic applications play an important role in low-input breeding strategies, such as that tagged “breeding without breeding” (El-Kassaby and Lstibůrek 2009; Wang *et al*. 2010; Lstibůrek *et al*. 2015). Among those, genomic selection is being applied to increase genetic gains in less time, to shorten breeding cycles with higher efficiency in resource usage (Isik *et al*. 2016; Cortés *et al*. 2020; Pégard *et al*. 2020), and to harness Mendelian effects by increasing production or disease resistance controlled by major genes (Merrick *et al*. 2021). Other genomics applications include the characterization of reproductive materials (e.g. Olsson *et al*. 2023), essential for an effective monitoring of improved material.

Mediterranean stone pine (*Pinus pinea* L.) is a good candidate to implement low-input breeding strategies because of its economic interest but limited importance in plantations. This species is widespread around the Mediterranean Sea, with a total current area exceeding 1 million hectares—if recent extensive plantations by private landowners are included. More than 250,000 ha of this species have been planted since 1990, especially in southern Portugal and western Turkey, linked to its edible seeds that are one of the most refined and expensive nuts in the world (Prada *et al*. 1997; Mutke *et al*. 2012, 2019). Stone pine breeding programmes have been hindered by the lack of genetic variability in the species. Earlier studies based on allozyme, chloroplast, and nuclear microsatellite markers, as well as candidate gene sequencing, reported limited genetic variation across the entire distribution range of the species (Fallour *et al*. 1997; Vendramin *et al*. 2008; Jaramillo-Correa *et al*. 2020). This lack of genome-wide genetic diversity has been attributed to an ancient and prolonged bottleneck, together with some biological attributes specific to this pine (i.e. seed not dispersed by wind and 3-year cone maturation; see Jaramillo-Correa *et al*. 2020).

Traditional breeding programmes of stone pine aiming at improving pine nut production started decades ago in Italy, Portugal, and Spain (Balguerías 1971; Magini and Giannini 1971; Carrasquinho *et al*. 2010). The strategy is based on plus tree selection and establishment of clonal archives to supply graft scions. Nut production has high clonal heritability, allowing effective selection in breeding programmes (Mutke *et al*. 2019). However, there are some constrains related to the costs of the establishment of clonal test for genetic evaluation by grafting, the large periods of time required for an efficient evaluation (up to 5 years after grafting for flower production and 3 years for cone development). There are also difficulties to provide a reliable identification of clones based on distinctive characters for registration according to the UE Directive 199/05. This difficulty is related to an absence of morphological or phenological distinctive descriptors (namely branching habit, crown form, leaf shape, or phenological calendar; Mutke *et al*. 2019), and a limited power of discrimination of the set of nuclear microsatellites (nSSRs) currently employed for clonal identification (Pinzauti *et al*. 2012). All of these constraints lead to a reduced number of clones available in the national breeding programmes. Currently, at the European level (see FOREMATIS database; https://ec.europa.eu/forematis/), a clonal mixture of 64 genotypes has been registered by the national authorities in Portugal, while 15 out of 390 clones have been registered in Spain.

To significantly push forward the breeding programme of stone pine using low-input strategies, we produced 2 datasets. (1) We genotyped 99 clones from the Spanish breeding programme, using the 4TREE multispecies Axiom´s microarray. (2) We phenotyped 2 commercially important traits (total number of cones unspoiled by pests and their weight) for the same clonal material measured in 3 clonal tests located in central and north-eastern Spain (10,563 to 15,124 records for the 2 traits, respectively). We had 3 main objectives to overcome the main constraints to deployment of improved material from the Spanish stone pine breeding programme. First, we assessed the potential identification of clones using the 4TREE SNP-array. Second, we estimated the genomic relationship matrix among the clones using the same markers. Third, we applied this information to develop a genomic prediction model for the 2 phenotypic traits. Finally, we provided insights to use this information in the context of the on-going breeding programmes of stone pine.

## Materials and methods

### Sampling

**Fig. 1.**
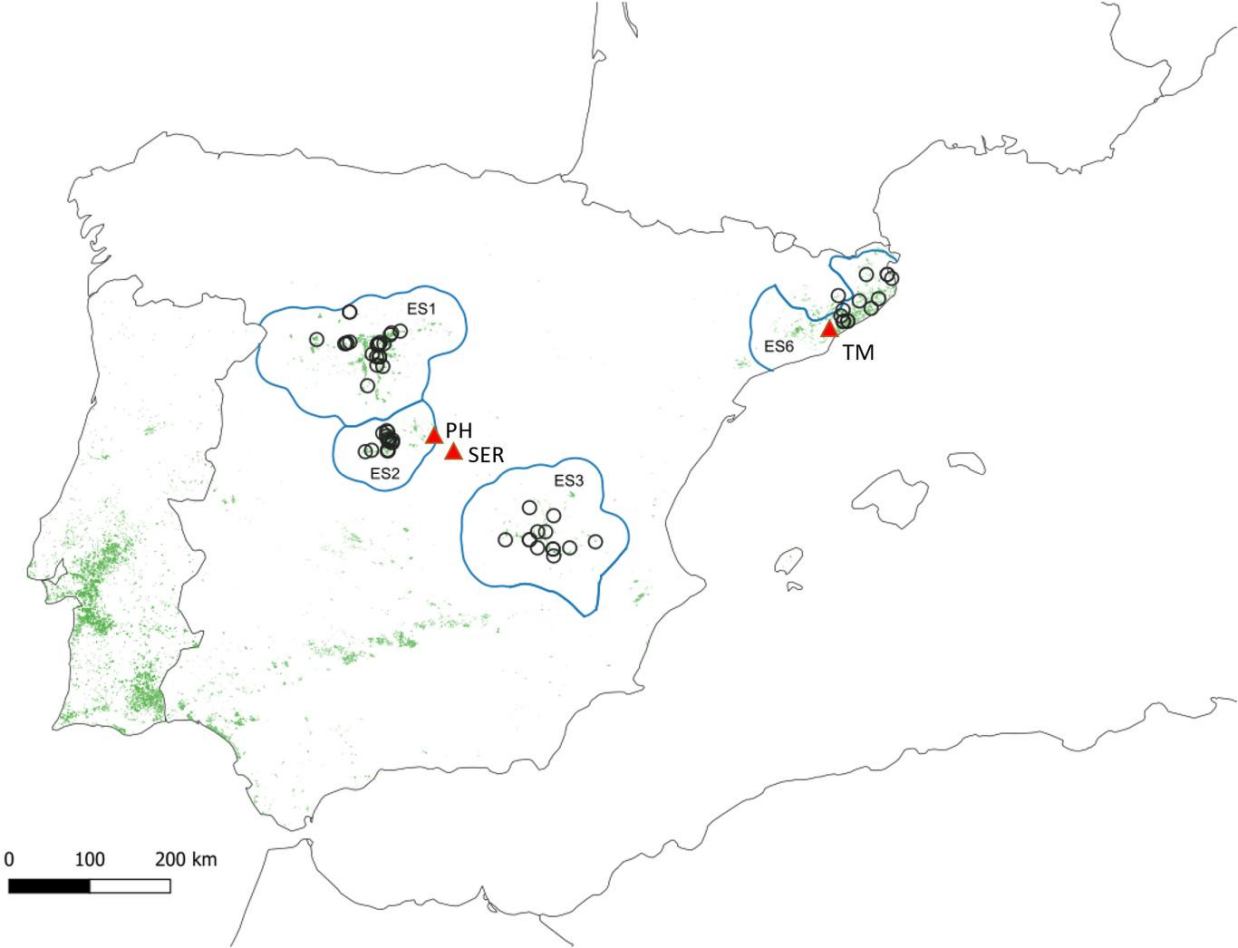
Location of 99 stone pine clones and the clonal test. Hollow circles: sampled clones; Red triangles: clonal tests (PH, Puerta de Hierro; SER, Serrranillo; TM, Torre Marimón); rasterized areas: stone pine natural distribution obtained from the Spanish forest map (MITECO 2015, miteco.gob.es); Blue line and label: sampled stone pine regions of provenance (modified from Prada *et al*. 1997; Marques *et al*. 2012).

We include data from 99 clones selected for cone production in the Spanish breeding programme (Supplementary File 1). These clones derive from 4 regions of provenance, as defined by Prada *et al*. (1997) for the marketing of forest reproductive material (FRM), namely ES1 (Northern Plateau, Douro basin), ES2 (Tiétar and Alberche Valleys, Spanish Central Range), ES3 (La Mancha), and ES6 (Coastal Catalonia) (Prada *et al*. 1997). Fifteen of these clones are registered (EU list of basic material) for the production of qualified reproductive material, with nut production being the main selection criterion (see Fig. 1 and Supplementary Table 1).

### Genotyping

We collected needles from 10 to 11 ramets (i.e. clonal replicates) of each of the 15 registered clones (*N* = 176), and from 1 ramet per clone for the remaining 84 unregistered clones, resulting in a total sampling effort of *N* = 260 (Supplementary Fig. A in File 1). Needles were dried in silica gel before DNA extraction using NucleoSpin Plant II Kit (Macherey-Nagel GmbH & Co. KG, Düren, Germany).

Samples were genotyped using the Axiom's 4TREE array (Guilbaud *et al*. 2020) at Thermo Fisher's Microarray Research Services Laboratory, Santa Clara (CA, USA). This array includes 5,671 SNPs for stone pine. The default Best Practices workflow was applied to call the SNPs in the Axiom Analysis Suite 5.0 software: All arrays with a dish quality control (dQC) value ≥0.82 and a QC call rate ≥97% were called, resulting in 2,245 SNPs, and 257 out of the 260 samples were successfully genotyped. The filtered data set were exported in both call code and VCF formats. Subsets of VCF files were created with *vcftools* v0.1.13 (Danecek *et al*. 2011). R version 4.1.2 was used for the subsequent statistical analyses (R Core Team 2022).

### Phenotyping

Two traits related to nut production were measured during multiple years in 3 clonal tests: Number of cones in the period analyzed (NC) and weight [MCW, measured in gram (g), as the mean cone fresh weight], estimated for the cones unspoiled by pests (i.e. larvae of *Dioryctria mendacella* or *Pissodes validirostris*). Diameter of the tree [DAG, in centimeter (cm)] was also measured as a covariate indicative of tree size.

The 3 clonal tests are located in Central Spain (PH, Puerta de Hierro; SER, Serranillo) and north-eastern Spain (TM, Torre Marimón) (Fig. 1, Table 1, Supplementary Table 2). Clonal material was obtained by grafting on 2-years-old plants. The clonal tests follow a block design, with different number of clones (56 out of the 99 are located in the 3 sites, with PH being the only site including all 99 clones), and planted at different years (PH: 1992–1998, SER: 2007, TM: 2008–2010). In the PH site, these traits were measured annually for 23 years (from age 5 to 28), while for the other 2 sites (SER and TM) the data correspond to 14 and 10 years, respectively, although some missing data are found (see Supplementary Table 3 for details). A total of 15,124 measurements were taken for NC and 10,563 for MCW at the 3 sites, and for the 99 genotyped clones.

**Table 1.** Description of stone pine clonal tests.

Site	Clonal test	Location*^a^*	Clones*^b^*	Ramets*^c^*	No. of crops measured/(age of trees)
PH	Puerta de Hierro	40°27′N/3°45′W	99 (312)	5.2	24/(age 5–28)
SER	Serranillo	40°40′N/3°10′W	62 (64)	6.0	13/(age 4–18)
TM	Torre Marimon	41°37′N/2°10′E	61 (64)	5.7	8/(age 5–12)

*^a^* Latitude/Longitude; *^b^*Number of genotyped clones (total number of clones) in the site; *^c^*Number of ramets per clone.

### Clone identification and genotyping error rate

Genetic diversity parameters of the 99 clones, namely observed and unbiased expected heterozygosity rates, were calculated using the R package *hierfstat* v0.5-11 (Goudet 2005). Minor allele frequencies (MAF) were obtained with *vcftools* v0.1.13. Consensus genotypes were created for each of the 15 registered clones (from the 4 to 11 confirmed ramets per clone), using a shell script (“awk” command) provided by Arun Seetharam (available at Github, https://github.com/aseetharam/awk_data_manipulation/blob/main/vcf_tricks.md). The number of mismatches between ramets and the consensus reference genotype were calculated with the function bitwise.dist from R package *poppr* v2.9.3 (Kamvar *et al*. 2014, 2015). Based on this information, coupled with a principal component analysis, each sample was either confirmed to belong to the expected clone, or excluded from the analyses. The number of mismatches was then re-calculated by comparing each ramet genotype to the corresponding consensus genotype. Per-sample genotyping error rates were estimated using this information, without considering missing data. After excluding samples with an error rate >2.5%, an additional genotyping error rate for each SNP was estimated based on all the surveyed ramets of the registered clones by dividing the number of mismatches by the total number of nonmissing genotypes for that specific SNP marker. The percentage of complete genotypes per loci and the percentage of complete genotypes per individual were computed by using the R package *adegenet* v2.1.5 (Jombart 2008).

Unique multilocus genotypes were collapsed into multilocus lineages using mlg.filter in *poppr* v2.9.3, applying the farthest neighbor clustering algorithm, Nei's distance and a similarity threshold, which was estimated to be 0.0399 using the cutoff_predictor function. Missing data were replaced with average allele counts. Any detected hidden duplicates among clones were interpreted to represent possible loss of identity preserved during vegetative multiplication cycles.

### Genomic prediction

A corrected phenotype value was estimated (clonal best linear unbiased prediction (BLUP)) for each clone within each trial for MCW and NC following 2 different mixed models: (1) since MCW data were a repeated measure, a single data point per ramet (ramet BLUP) was estimated through a linear mixed model, correcting by calendar year (as random factor) given that cone production is severely affected by masting; (2) in a different approach for NC, a single data point for each ramet was estimated as the total sum of produced cones across all the years, controlling prior summation by calendar year, and DAG at that year (to correct for allometric effects; see Supplementary Table 3 for additional information). Once such summation was done, each ramet had a single observation of total yield of number of cones and numbers of years measured. Subsequently, having the estimation of MCW and NC at ramet level, clonal BLUPs were estimated by means of linear models, using number of years measured as correcting covariate only for NC phenotype. From similar models, we also calculated corrected estimations of clonal broad-sense heritability (*H*^2^; more details in Supplementary File 2).

To perform genomic prediction, the following model was used:Y=Xb+Za+e,where **Y** is the vector of phenotypic ramet level traits; **b** is a vector of fixed effects (including the general mean); **a** is a normally distributed vector of individual random genetic effects [**a** ∼ N(0, genetic relationship matrix (GRM) *σ*_g_^2^)], where GRM is a genomic relationships matrix among individuals and *σ*_g_^2^ is the genetic variance; and **e** is a normally distributed vector representing the random residual effects [**e** ∼ *N*(0, *I σ*_e_^2^)], where *I* is an identity matrix and *σ*_e_^2^ the residual variance. GRM was computed following Endelman and Jannink (2012) as the mean probability of identity by state across loci, by using the R package *rrBLUP* v4.6.3 (Endelman 2011). The diagonal element of this matrix has a mean that equals 1 + *f*, where *f* is the inbreeding coefficient [i.e. the probability that the 2 alleles at a randomly chosen locus are identical by descent (IBD) from the base population; Endelman and Jannink 2012]. In addition, the inbreeding coefficient *F*_IS_ [calculated as 1 − (Ho/Hs)] and its 95% C.I. were calculated with *hierfstat* v0.5-11 (Goudet 2005). A heatmap and dendrogram of IBD values were obtained with the function gl.grm and a network diagram with gl.grm.network (relatedness_factor = 0.0001) from R package *dartR* v2.9.7 (Gruber *et al*. 2018).

SNP effects were predicted for each individual trait using R package *rrBLUP* (Endelman 2011), and solved by restricted maximum likelihood. Predictive ability (*r*_y_) was estimated per trait as the Pearson's correlation coefficient between the observed clonal BLUPs and the genomic-estimated breeding values using cross-validation (CV) (Müller *et al*. 2018; Bouvet *et al*. 2020). We performed a 9-fold CV for each of the 3 sites.

## Results

### Clonal identification and genotyping error rate

**Fig. 2.**
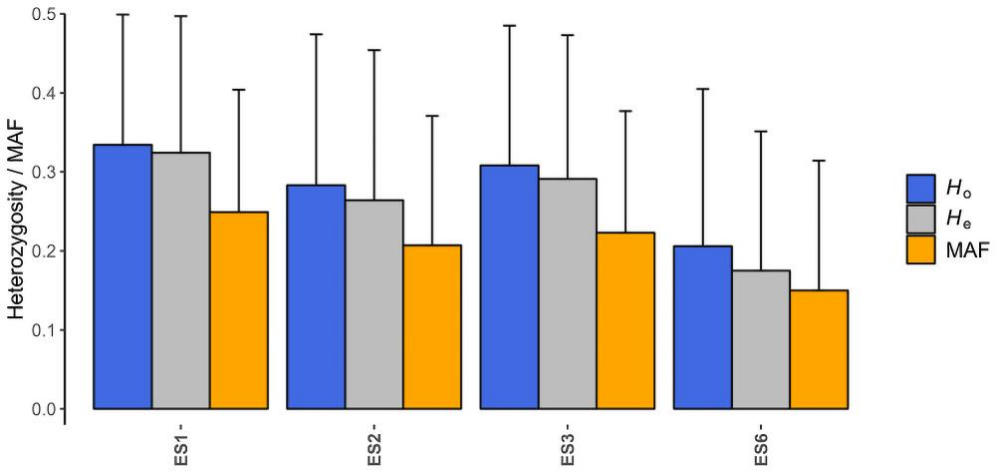
Observed (*H*_0_), expected (*H*_e_) heterozygosity and MAF for 99 stone pine clones of the Spanish breeding programme, from 4 regions of provenance (ES1, ES2, ES3, and ES6).

The SNP-array defined a unique genotype for each of the 99 clones. The heterozygosity values and MAF for these clones across the different regions of provenance indicate high genetic variation of these markers in the sampled genotypes, suitable for clonal identification (Fig. 2).

For the 15 registered clones, 16 samples were excluded from the downstream analyses (9.2%) as they did not match the SNP profile of their supposed clone (Table 2). The proportion of complete genotypes per locus (2,245 SNPs) ranged from 0.884 to 1, the average being 0.989. In general, the proportion of complete genotypes per individual was high, the lowest value being 0.910. The mean number of mismatches within clone ranged from 3 to 33, corresponding to a genotyping error rate ranging from 0.001 to 0.014. The SNP error rate was low as only 272 markers (out of 2,245) presented a genotyping error rate >1% (Supplementary Fig. 1).

**Table 2.** Statistics related to the genotyping of the 15 Spanish registered clones of stone pine.

Clone	Nb GT	Proportion of SNPs	Mismatches	Error rate	Region of provenance
c1011	7	0.970	3	0.001	ES1
c1012	5	0.959	4	0.002	ES1
c1073	12	0.965	15	0.006	ES1
c1123	12	0.959	10	0.004	ES1
c1201	11	0.958	11	0.005	ES1
c2004	10	0.948	33	0.014	ES2
c2048	13	0.960	13	0.006	ES2
c2068	10	0.952	28	0.012	ES2
c3029	11	0.962	13	0.005	ES3
c3048	12	0.955	11	0.005	ES3
c3057	11	0.955	9	0.004	ES3
c3063	11	0.961	14	0.006	ES3
c6010	11	0.960	12	0.005	ES6
c6015	10	0.957	8	0.004	ES6
c6053	12	0.967	6	0.003	ES6

Mean values for each genet, calculated from several ramets, are provided. Nb GT, number of genotypes used in the analysis.

### Genomic prediction

**Fig. 3.**
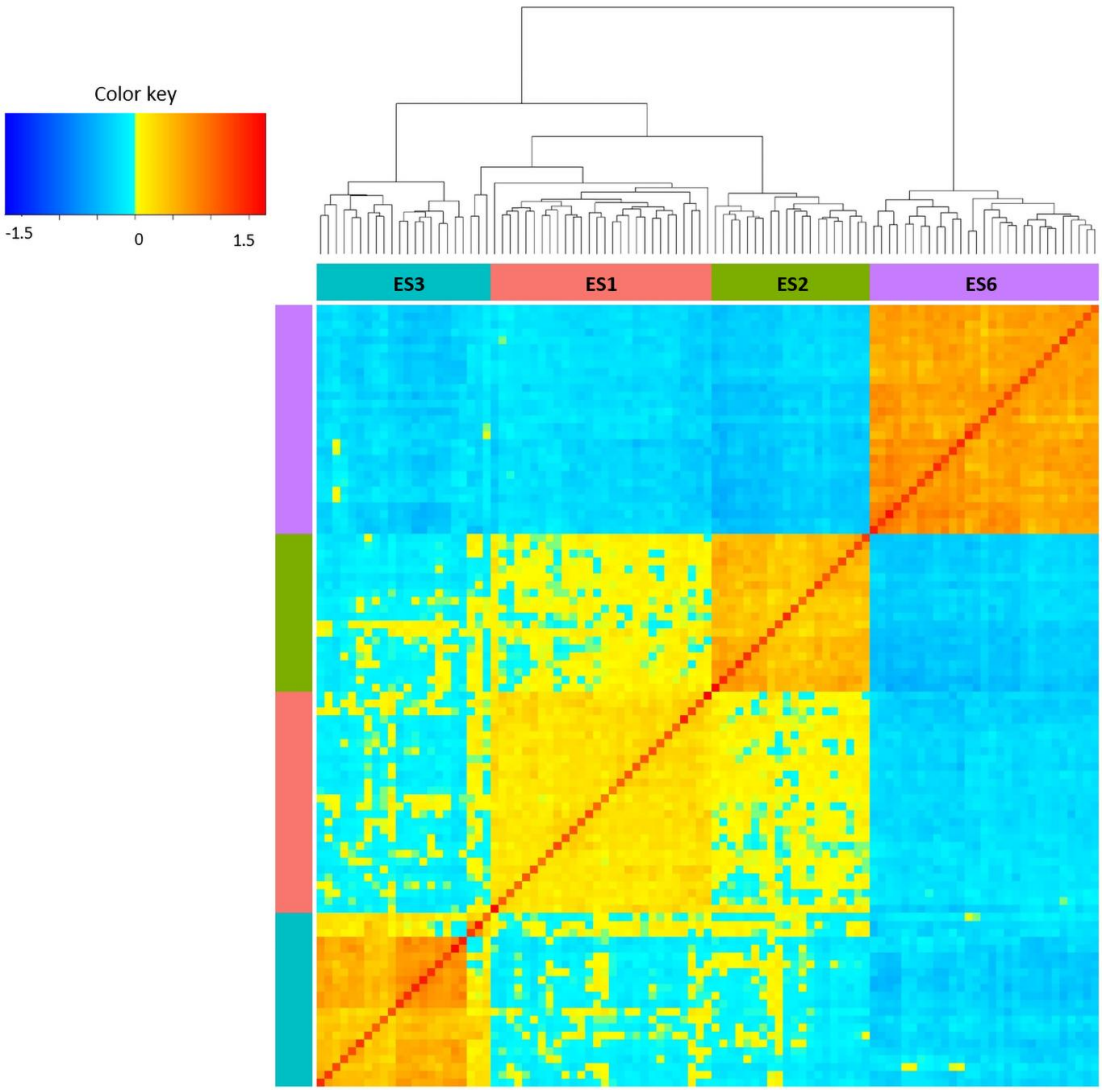
Heatmap and dendrogram of the additive GRM of 99 clones belonging to 4 regions of provenance (ES1, ES2, ES3, and ES6) based on 2,245 genome-wide SNP markers. The darker the cold color in the heat map color, the lower the additive genetic relationship between 2 clones, and the darker the warm color, the higher the genetic relationship between 2 clones.

The heatmap and dendrogram of the GRM matrix reflect a population genetic structure that mirrors the grouping clones according to their region of provenance. The only exception was 3 clones from the region ES3 that grouped together with ES1 clones (Fig. 3). The trees from Catalonia (ES6) were placed in a more distant branch than those from the other 3 provenance regions from inner Spain.

The network representation of the IBD shows strong relationships among individuals from ES6, none for individuals from ES1, and an intermediate situation for ES2 and ES3 (Supplementary Fig. 2). Genomic relationships of the four regions were summarized by their inbreeding coefficients (derived from the diagonal of the GRM matrix) and mean values of IBD with respect to the other regions (i.e. individuals from the same region are excluded) (Table 3).

**Table 3.** Inbreeding coefficient (and SD) for the 4 stone pine provenance regions included in the analysis, together with mean *F*_IS_ values (and C.I.) and mean IBD values (and SD) of individuals from each region relative to the same and the other regions.

Region	N1	Inbreeding from IBD matrix	FIS	IBD with clones from the same region	N2	IBD with clones from other regions
ES1	32	0.010 (0.180)	0.029 (0.020–0.037)	0.190 (0.070)	71	−0.099 (0.137)
ES2	20	0.223 (0.135)	0.066 (0.056–0.077)	0.011 (0.090)	83	−0.116 (0.180)
ES3	22	0.167 (0.175)	0.053 (0.043–0.065)	0.432 (0.223)	81	−0.129 (0.131)
ES6	29	0.506 (0.136)	0.151 (0.138–0.164)	0.653 (0.108)	74	−0.265 (0.094)
Total	103	0.230 (0.251)	0.075	0.315 (0.272)	309	−0.152 (0.152)

N1, number of clones in the region; N2, number of clones used in comparison with clones from the other regions.

Corrected clonal heritability (*H*^2^) ranged from 0.21 to 0.52 for the 2 traits (NC and MCW) and the 3 clonal tests (PH, SER, and TM). They were slightly lower for MCW, ranging from 0.21 at PH test to 0.40 at TM test, once the variance of calendar year was removed. For NC, values varied from 0.24 at TM test to 0.52 at SER test.

There are some promising clones for selection based on the BLUP values for the 2 traits (NC and MCW) measured in the PH clonal test where all the 99 clones have been evaluated in the same environment (Table 4). The 10% of the top-ranking clones (see Supplementary Table 4) are clearly superior to the mean value in the clonal test (65% for NC and 20% for MCW) and to the 15 clones already registered (20 and 3%, respectively). These 10% top clones include 6 out of the 15 registered clones (c1011, c1012, c1201, c1073, c1123, and c3048).

**Table 4.** BLUPs values (and SD) for the 2 traits (NC and MCW) considered in the analysis in the PH clonal test (99 clones represented in the same environment).

	*N*	NC	MCW
Mean	99	31.26 (9.75)	267.61 (30.27)
Registered clones	15	37.41 (9.92)	276.58 (29.06)
10% top clones	10	51.67 (4.74)	321.67 (10.35)

*N*, number of clones.

**Fig. 4.**
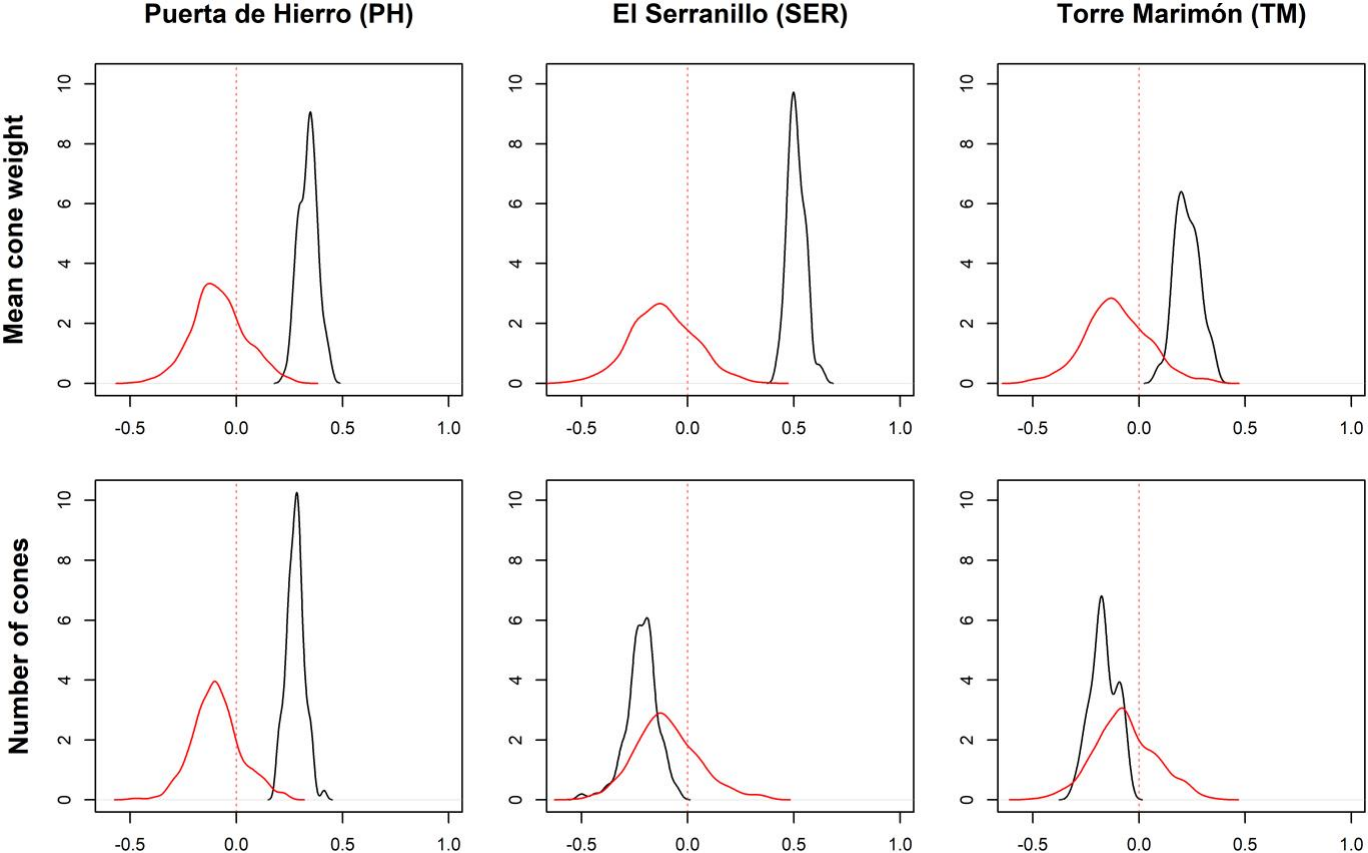
Density plots for predictive ability (*r*_y_) based on 9-fold cross-validation for 2 traits (mean cone weight and number of cones) and 3 sites (PH, SER, and TM).

Predictive ability (*r*_y_) for NC in the PH clonal test was considerable, in the low to moderate range (0.278 ± 0.042; Fig. 4). For the other 2 clonal tests, *r*_y_ values for this trait were not significantly different from the null distribution of permutated values. However, for the MCW trait, prediction accuracies were substantial and significantly different from the null distribution in all the 3 clonal tests (0.227 ± 0.058, 0.338 ± 0.044, and 0.511± 0.042 for TM, PH, and SER, respectively). Importantly, *r*_y_ depends on the number of clones tested, explaining the higher values of *r*_y_ for NC in the PH clonal test, where all the clones were represented.

## Discussion

This study highlights the crucial importance of developing genomic and phenotyping tools for paving the road toward more effective breeding strategies in stone pine. Thanks to extensive genomic and phenotypic datasets, we are now able to: (1) identify clones from the Spanish breeding programme, a determinant step for tracking samples from their production to their commercialization, (2) estimate the genetic relatedness among clones, an essential information for low-input breeding and genomic selection, and (3) apply this information to genomic prediction for relevant commercial traits such as mean cone weight.

### Clonal identification and genotyping error rate

Genotyping several independent clonal replicates (ramets) allowed the estimation of a reliable genotyping error rate for stone pine with the 4TREE SNP-array. Error rates (from 0.1 to 1.4%, with a mean of 0.5%) were similar to those obtained in *Pinus sylvestris* (0.9%; Kastally *et al*. 2022), but higher than in *Pseudotsuga menziesii* (0.04%; Howe *et al*. 2020). Differences in ramet genotypes due to somatic mutations were not expected, as they should be very rare in stone pine due to its slow mutation rate (Vendramin *et al*. 2008). The proportion of successful SNP genotyped in conifers is, in general, lower than in other plants (Howe *et al*. 2020) and, therefore, relaxed thresholds have often been applied to increase the number of available SNPs (Howe *et al*. 2020; Kastally *et al*. 2022). However, a larger number of SNPs comes at the cost of lower accuracy, so this option may not be appropriate for all applications. For species with low genetic variation, such as stone pine, the consistency and accuracy of SNP genotyping ensure that the results are reliable. Therefore, a higher accuracy of the SNPs is preferred to a higher number of SNPs for clonal identification of highly similar clones.

In the present study, the erroneously assigned ramets in Spanish clonal banks for production of qualified reproductive material (10.1%) highlights the operational problems involved in warranting the genetic identity of FRM after repeated cycles of nursery handling, grafting, and outplanting. Consequently, we advocate for additional genotyping in the clonal banks to confirm or discard other possible sampling or handling errors, and thereby accurately certify the production of qualified material. This substantial error rate in assignment also highlights the need for informative markers in a species with a previously reported low level of polymorphism in its genome and a lack of morphological or phenological distinctive characters among clones.

The set of SNPs provides an accurate and reliable identification of existing and future selected stone pine clones from the breeding programme. At present, the existing nSSR markers (Pinzauti *et al*. 2012) do not allow the identification of superior trees selected in the clonal tests included in our study (MITECO, internal report). Moreover, nSSR markers present a high degree of homoplasy (Guichoux *et al*. 2011), which limits an accurate identification of clones in a species with low level of genetic diversity. In genetically homogeneous species like stone pine, the reduced genetic variation can pose challenges when attempting to differentiate individuals or populations. The high discriminatory power of a large number of SNPs allows to pinpoint variations at specific loci, even when the overall genetic diversity is limited. In addition, biallelic SNPs are suitable for high-throughput genotyping and are more straightforward to score, compared to multiallelic nSSRs. The 4TREE SNP-array allows the characterization of the existing clones, and our genomic prediction output suggest that new clones may outperform the previously registered ones, broadening thereby the genetic base of the breeding programme.

### Genomic prediction

The inbreeding coefficients estimated from the relationship matrix in stone pine clones from different provenance regions (ranging from 0.010 to 0.506) were higher than the *F*_IS_ estimates (ranging from 0.029 to 0.151). The values of inbreeding are similar to those reported using 12 nSSR in 4 natural populations of stone pine (0.080–0.413) (Pinzauti *et al*. 2012). Several methodologies exist to compute the GRM and consequently, different inbreeding estimates might provide incongruent measures (Villanueva *et al*. 2021). Inbreeding affects, linearly, the additive component of genetic variance and the expected value of quantitative traits, which is why it is important to estimate this parameter. In maritime pine (*Pinus pinaster* Aiton), number of cones is the trait with the highest inbreeding depression in comparison to others related to growth and straightness (Durel *et al*. 1996), causing a reduction in cone production, growth, or straightness. Forest trees are typically outbreeders and carry relatively high genetic loads (deleterious alleles) to avoid inbreeding, which is also the case in stone pine (Jaramillo-Correa *et al*. 2020). The practical importance of selfing is generally low, owing to mechanisms that effectively limit fertilization with self-pollen (in conifers, embryonic lethals) (Mátyás 2004). Nevertheless, the actual level of inbreeding should not represent any problem in the short-term in most of the populations analyzed, except ES6. In this population, the clones show a higher degree of genetic relationship among them as indicated by the mean values of IBD. The evaluation of cone production in each generation as a major selection trait, and the estimated relationships among selected genotypes, would allow the efficient management of inbreeding by managing genetic contributions over generations (Sánchez *et al*. 2003).

The additive GRM opens new avenues to identify association of genotypes with traits of interest, provided an adequate sample size. There is a clear structure to the relationships among the clones from different populations and we should therefore take this information into account when advancing in the breeding programme of the species. Genetic relationship matrices can also be used to partition the additive component of variance and, thus, estimate narrow-sense heritability using an animal model (White *et al*. 2007). The estimation of breeding values and genetic parameters (e.g. heritability, G–E interaction, and genetic correlations), in combination with the precise identification of all the clones included in our study, opens the possibility of selecting new clones to be included in the register of material for commercialization. Indeed, the 15 clones registered in Spain were not the very best performing for any of the 2 traits, among all the evaluated clones in the clonal tests (Guadaño and Mutke 2016). Here, we have identified new promising clones for cone production, which would allow genetic gains of the order of 65% for NC and 20% for MCW (in comparison to 20 and 3% for the registered clones). However, the results need to be confirmed with a larger number of genotypes as there is a risk of overfitting the prediction model due to the rather small number of genotypes. Once confirmed, these new clones can be registered, included in the Spanish breeding programme and tested for their superior qualities in different environments according to the legislation.

These new clones will be used as basic material in clonal banks to produce tested material. Usually, in stone pine vegetative material is obtained by grafting to establish productive cone plantations. But the new clones could be used to establish progeny tests (based in open pollinated seeds collected in the existing clonal banks), using genomic prediction for early selection to select the best performing seedlings. However, we need to explore more thoroughly the genomic prediction models, and especially in relation with the effect of number of clones, the gene-environment interaction across robust multisite clonal tests and the accuracy of the planned cross-generation genomic prediction.

Genomic prediction models show contrasting output depending on the considered trait, with predictive ability for MCW being substantial in all the 3 clonal tests despite the reduced sampling sizes, while that for NC being also substantial in the PH clonal test but nonsignificant in SER and TM. Some of the differences are related to contrasting environmental factors in the clonal test among years. To prevent the effect of extreme crop years, our genomic prediction is based on the mean production of cones over a long period. The values obtained for MCW are in the lowest ranges compared to those obtained in maritime pine (Bartholomé *et al*. 2016; Isik *et al*. 2016) and loblolly pine (*Pinus taeda* L.; Zapata-Valenzuela *et al*. 2013), while higher than in white spruce (*Picea glauca*; Lenz *et al*. 2020a). We also detected differences among the sites, which could have been caused by the different number of clones tested (i.e. higher number of clones in PH compared to SER and TM). Overall, genomic selection is a very promising approach in species such as stone pine, which lacks the industrial critical mass to establish an extensive breeding programme (Grattapaglia 2022), and in which phenotyping is costly in terms of time, space, and human resources. Our results open the field for future evaluation of genetic tests or provenance tests for new traits of interest in stone pine, related to adaptation to abiotic hazards (e.g. drought) and new emerging pests and diseases (e.g. *Leptoglossus occidentalis* or *Toumeyella parvicornis*; Garonna *et al*. 2018), similar to what has been applied in other forest tree species (El-Dien *et al*. 2016; Ukrainetz and Mansfield 2020; Lenz *et al*. 2020b; Laverdière *et al*. 2022).

### Conclusion

By assessing a clonal collection of stone pine trees with a suitable genomic tool and extensive phenotyping, we provided guidance for future breeding programmes. We first demonstrated that clones could be reliably identified, including unregistered clones that were previously indistinguishable by using other molecular markers. The generated consensus genotypes of the 15 registered clones together with those of the 84 additional clones will serve as the first reference entries in the stone pine breeding programme database (available at Zenodo with DOI: 10.5281/zenodo.8185591), which will expand in the future as more clones are registered. We then reconstructed genomic relationships, showing that there is a congruent pattern between the relatedness and the provenance of the samples. Finally, we demonstrated that genomic prediction models are useful for commercial traits targeted by the stone pine breeding programme, and our output open the field for exploration of new traits of interest related to adaptation under future climatic conditions and emerging pests and diseases, with new material currently being evaluated in existing clonal and provenance tests. By applying genomic prediction models, we can reduce the effort and time for selection (as we can make a first selection based on the genotypes of seedlings from the clone trials), together with the cost of genetic testing, as only those selected seedlings and the controls should be installed in genetic tests.

## Supplementary Material

jkaf056_Supplementary_Data

## Data Availability

The data sets analyzed in the current study are available at Zenodo with DOI: 10.5281/zenodo.8185591 and include (1) genotypes of 257 samples from 99 clones; and (2) phenotypes used in the study. Supplemental material available at G3 online.
