# Supplementary material for: Low-input breeding potential in stone pine, a multipurpose forest tree with low genome diversity
Source: G3 (Bethesda). 2025 Mar 12;15(5):jkaf056. doi: 10.1093/g3journal/jkaf056 (PMC12060235; doi:10.1093/g3journal/jkaf056)

Supplementary Figure S1. Error rates based on ramets from 15 registered *P. pinea* clones and 2,245 SNPs. Per SNP error rate calculated by dividing the number of samples with a mismatch by the total number of samples without mismatches.

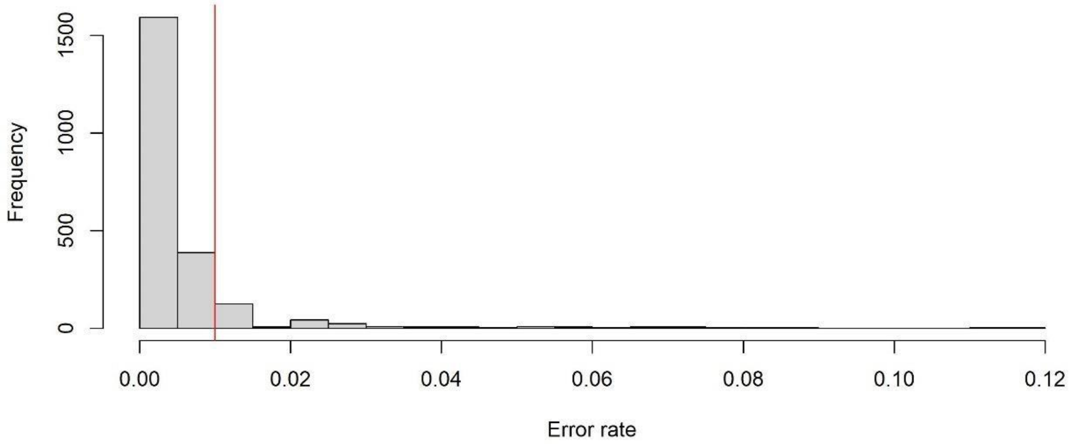

Supplement: jkaf056_Supplementary_Data [file jkaf056_supplementary_data.zip › Figure_S1_G3-2024-405456.pdf]
