# Supplementary material for: Low-input breeding potential in stone pine, a multipurpose forest tree with low genome diversity
Source: G3 (Bethesda). 2025 Mar 12;15(5):jkaf056. doi: 10.1093/g3journal/jkaf056 (PMC12060235; doi:10.1093/g3journal/jkaf056)

Supplementary Figure S2. Network diagram based on IBD values of the of 99 stone pine clones applying a very low relatedness factor of 0.0001 (Fruchterman-Reingold layout).

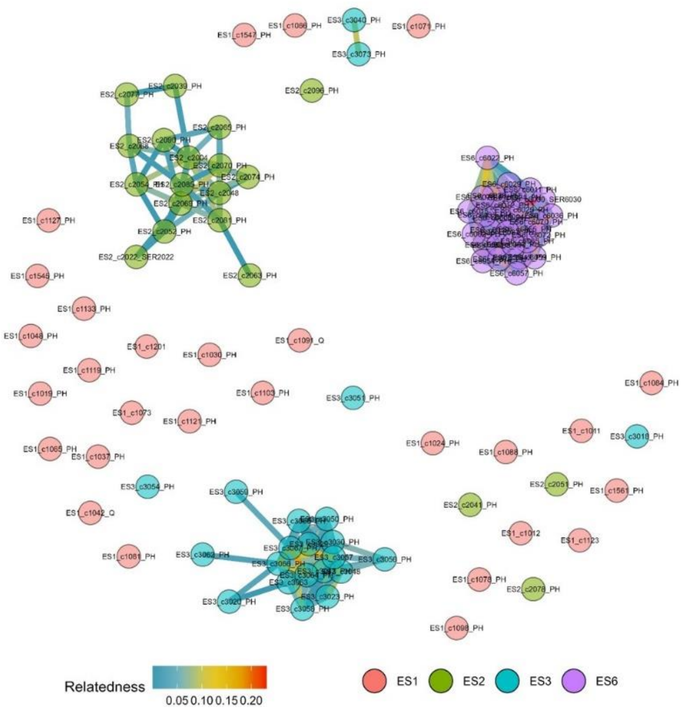

Supplement: jkaf056_Supplementary_Data [file jkaf056_supplementary_data.zip › Figure_S2_G3-2024-405456.pdf]
