# Supplementary material for: Low-input breeding potential in stone pine, a multipurpose forest tree with low genome diversity
Source: G3 (Bethesda). 2025 Mar 12;15(5):jkaf056. doi: 10.1093/g3journal/jkaf056 (PMC12060235; doi:10.1093/g3journal/jkaf056)
Supplement: jkaf056_Supplementary_Data [file jkaf056_supplementary_data.zip › File_S1_G3-2024-405456.pdf]

Supplementary File S1. A) Sampling scheme.

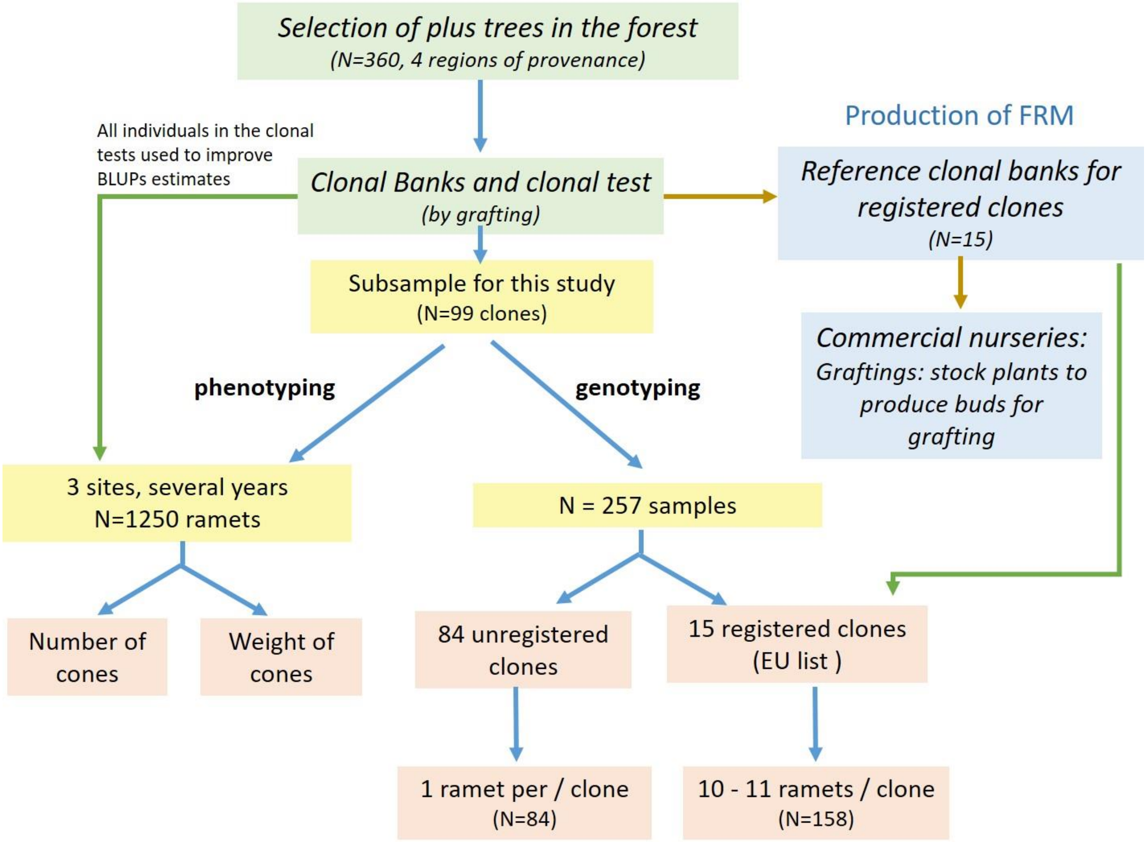

B) Sample sizes used in the study.

Objective	N	Description
Error rate and clonal identification	145*	15 registered clones, Spain. 4 regions of provenance
Genetic relationship matrix	99	Clones from the Spanish breeding population.
Genomic prediction	99	Genotypes of 99 clones and associated phenotypes (1250 ramets) in 3 clonal tests.

*Number of verified genotypes: 158 (as some putative ramets were genotyped several times for quality control)
