# Supplementary material for: Low-input breeding potential in stone pine, a multipurpose forest tree with low genome diversity
Source: G3 (Bethesda). 2025 Mar 12;15(5):jkaf056. doi: 10.1093/g3journal/jkaf056 (PMC12060235; doi:10.1093/g3journal/jkaf056)
Supplement: jkaf056_Supplementary_Data [file jkaf056_supplementary_data.zip › File_S2_G3-2024-405456.pdf]

Supplementary File S2. Linear models used to estimate ramet values, clonal BLUPs and broad-sense heritabilities.

RAMET VALUES:

For MCW:

MCW = Calendar year + Ramet ID + Residuals

Getting BLUPs of Ramet ID

For NC:

Firstly, estimating Accumulated NC per ramet, after correction of yearly measurements by Calendar year and Diameter of the tree. Then:

Accumulated NC = Number of years measured + Residuals

Getting Residuals as corrected Ramet phenotypes

CLONAL BLUPS:

For MCW:

Ramet BLUPS = Clone ID + Residuals

For NC:

Corrected Ramet Phenotypes = Clone ID + Residuals

HERITABILITIES:

For MCW:

MCW = Calendar year + Clone ID + Ramet ID (nested in Clone ID) + Residuals

H^2 = VAR (Clone ID) / (VAR (Clone ID) + VAR (Residuals))

For NC:

Corrected Ramet Phenotypes = Clone ID + Residuals

H^2 = VAR (Clone ID) / (VAR (Clone ID) + VAR (Residuals))

Random factors: Calendar year, Ramet ID, and Clone ID

Fixed factors: Number of years measured

VAR (X): variance of X factor
