# Supplementary material for: Low-input breeding potential in stone pine, a multipurpose forest tree with low genome diversity
Source: G3 (Bethesda). 2025 Mar 12;15(5):jkaf056. doi: 10.1093/g3journal/jkaf056 (PMC12060235; doi:10.1093/g3journal/jkaf056)
Supplement: jkaf056_Supplementary_Data [file jkaf056_supplementary_data.zip › Table_S1_G3-2024-405456.pdf]

Supplementary Table S1. Number of sampled, genotyped and verified ramets and origin of the 15 registered Spanish stone pine clones.

Clone	Nb sampled	Nb succ. genotyped	Nb GT (incl. replicates)	Nb ind (ramets)	Region of provenance	Population (Province)
c1011	11	10	7	6	ES1, Northern Plateau	Portillo (Valladolid)
c1012	11	10	5	4	ES1, Northern Plateau	Portillo (Valladolid)
c1073	10	10	12	10	ES1, Northern Plateau	Tordesillas (Valladolid)
c1123	11	10	12	11	ES1, Northern Plateau	Iscar (Valladolid)
c1201	11	11	11	11	ES1, Northern Plateau	not located (Valladolid)
c2004	11	11	10	10	ES2, Tiétar and Alberche Valleys	Hoyo de Pinares (Avila)
c2048	11	11	13	11	ES2, Tiétar and Alberche Valleys	Almorox (Toledo)
c2068	11	11	10	10	ES2, Tiétar and Alberche Valleys	San Martín de Valdeiglesias (Madrid)
c3029	10	10	11	10	ES3, La Mancha	El Provencio (Cuenca)
c3048	11	11	12	11	ES3, La Mancha	Pozoamargo (Cuenca)
c3057	11	11	11	10	ES3, La Mancha	Casas de Haro (Cuenca)
c3063	11	11	11	10	ES3, La Mancha	El Picazo (Cuenca)
c6010	10	10	11	10	ES6, Coastal Catalonia	Sta. Coloma de Farnes (Girona)
c6015	10	10	10	10	ES6, Coastal Catalonia	Llagostera (Girona)
c6053	11	11	12	11	ES6, Coastal Catalonia	Dosrrius (Barcelona)

Nb sampled: number of individuals that were collected and sent for genotyping. **Nb succ. genotyped**: number of successfully genotyped individuals. **Nb GT:** number of verified genotypes of this clone (some individuals were genotyped several times for quality control); **Nb ind:** number of verified individuals (i.e. ramets) of this clone
