# Supplementary material for: Low-input breeding potential in stone pine, a multipurpose forest tree with low genome diversity
Source: G3 (Bethesda). 2025 Mar 12;15(5):jkaf056. doi: 10.1093/g3journal/jkaf056 (PMC12060235; doi:10.1093/g3journal/jkaf056)
Supplement: jkaf056_Supplementary_Data [file jkaf056_supplementary_data.zip › Table_S2_G3-2024-405456.pdf]

Supplementary Table S2. Description of the clonal tests.

Site	PH	PH	PH	PH	SER	TM
**Clonal Bank**	B23PH1	B23PH2	B23PH3	B23PH4	B23SER	B23IRTA-P435
**Institution**	MITECO	MITECO	MITECO	MITECO	MITECO	IRTA
**Location**	Puerta de Hierro, M	Puerta de Hierro, M	Puerta de Hierro, M	Puerta de Hierro, M	El Serranillo, GU	Torre Marimón, B
**Grafting year**	1992	1993	1994	1998	2007	2008to 2010
**Design**	BC	BC	BC	BC	BI	BC
**Spacing**	3x3	3x3	3x3	5x5	6x6	6x6
**# Replicates**	7	4	4	4	6	3x2
**# Grafts**	593	478	260	289	288	367
**# Clones**	90	90	78	73	48	64
**# ramets/clon**	6.6	5.3	3.3	4.0	6	5.73
**ha**	0.6	0.7	0.6	0.8	1.0	1.0
