# Supplementary material for: Low-input breeding potential in stone pine, a multipurpose forest tree with low genome diversity
Source: G3 (Bethesda). 2025 Mar 12;15(5):jkaf056. doi: 10.1093/g3journal/jkaf056 (PMC12060235; doi:10.1093/g3journal/jkaf056)
Supplement: jkaf056_Supplementary_Data [file jkaf056_supplementary_data.zip › Table_S3_G3-2024-405456.pdf]

Supplementary Table S3. Number of trees phenotyped for all the 315 clones of the Breeding programme in each site and for the 99 clones that have been genotyped.

		PH		SE		TM			
**Age**	**Trait**	**Total**	**Genot.**	**Total**	**Genot.**	**Total**	**Genot.**	**Total**	**Genotyped**
4	NC							256	245
	MCW			184	176			184	176
5	NC	1573	510			359	341	1932	851
	MCW	1200	397			289	273	1489	670
6	NC	1573	510	256	245	331	315	2160	1070
	MCW	1254	414	199	189	189	179	1642	782
7	NC	1573	510	408	397	359	341	2340	1248
	MCW	1102	359	140	140	221	211	1463	710
8	NC	1573	510	256	245	359	341	2188	1096
	MCW	1249	420			332	316	1581	736
9	NC	1573	510	408	397	361	343	2342	1250
	MCW	906	297	406	395	361	343	1673	1035
10	NC	1573	510	408	397	361	343	2342	1250
	MCW	1133	353	256	245	361	343	1750	941
11	NC	1375	465	256	245	361	343	1992	1053
	MCW	1296	437	256	245	360	342	1912	1024
12	NC	1375	465	408	397	286	271	2069	1133
	MCW	883	284	289	278	286	271	1458	833
13	NC	1269	441	408	397			1677	838
	MCW	1039	354	257	251			1296	605
14	NC	1050	410	152	152			1202	562
	MCW	645	237	152	152			797	389
15	NC	1050	410	408	397			1458	807
	MCW	584	230	399	388			983	618
16	NC	1050	410	152	152			1202	562
	MCW	565	225	99	99			664	324
17	NC	836	359					836	359
	MCW	542	239					542	239
18	NC	836	359	151	151			987	510
	MCW	392	159	149	149			541	308
19	NC	836	359					836	359
	MCW	490	218					490	218
20	NC	586	271					586	271
	MCW	117	61					117	61
21	NC	759	325					759	325
	MCW	516	210					516	210
22	NC	694	318					694	318
	MCW	229	106					229	106
23	NC	397	203					397	203
	MCW	150	76					150	76
24	NC	397	203					397	203
	MCW	193	91					193	91
25	NC	397	203					397	203
	MCW	222	110					222	110
26	NC	397	203					397	203
	MCW	309	153					309	153
27	NC	269	132					269	132
	MCW	232	111					232	111
28	NC	136	73					136	73
	MCW	81	37					81	37
**Total**	**NC**	**23,147**	**8,669**	**3,927**	**3,817**	**2,777**	**2,638**	**29,851**	**15,124**
**Total**	**MCW**	**15,329**	**5,578**	**2,786**	**2,707**	**2,399**	**2,278**	**20,514**	**10,563**
