# Supplementary material for: Low-input breeding potential in stone pine, a multipurpose forest tree with low genome diversity
Source: G3 (Bethesda). 2025 Mar 12;15(5):jkaf056. doi: 10.1093/g3journal/jkaf056 (PMC12060235; doi:10.1093/g3journal/jkaf056)
Supplement: jkaf056_Supplementary_Data [file jkaf056_supplementary_data.zip › Table_S4_G3-2024-405456.pdf]

Supplementary Table S4. BLUPs estimates of the 10 best performing clones in PH genetic trial for each of the two traits and overall mean values (*N* = 99).

Clone Code	NC	Clone Code	MCW
**c2081**	59.699	**c3067**	340.534
**c1061**	56.161	**c1103**	330.700
**c1065**	55.896	**c3058**	329.394
**c1011**	53.585	**c3018**	327.789
**c1201**	51.813	**c6021**	324.681
**c3018**	51.520	**c3048**	314.985
**c1012**	48.933	**c1123**	314.186
**c1071**	48.074	**c1078**	313.029
**c6047**	45.593	**c2063**	312.407
**c2085**	45.457	**c1073**	309.037
**Mean**	31.262		267.606
